# RETRACTION: Effects of Enhanced Recovery After Surgery Nursing Program on the Surgical Site Wound Infection and Postoperative Complications in Patients of Gastric Cancer: A Meta‐Analysis

**DOI:** 10.1111/iwj.70440

**Published:** 2025-03-26

## Abstract

**RETRACTION:** SunC.‐Z. , LiX.‐X. , CaoS.‐H. , LiW. , LiN.‐M. , “Effects of Enhanced Recovery After Surgery Nursing Program on the Surgical Site Wound Infection and Postoperative Complications in Patients of Gastric Cancer: A Meta‐Analysis,” International Wound Journal 21, no. 2 (2024): e14687, 10.1111/iwj.14687.PMC1131054439118459

The above article, published online on 31 January 2024, in Wiley Online Library (http://onlinelibrary.wiley.com/), has been retracted by agreement between the journal Editor in Chief, Professor Keith Harding; and John Wiley & Sons Ltd. Following an investigation by the publisher, all parties have concluded that this article was accepted solely on the basis of a compromised peer review process. The editors have therefore decided to retract the article. The authors did not respond to our notice regarding the retraction.

**RETRACTION:** C.‐Z. Sun , X.‐X. Li , S.‐H. Cao , W. Li , N.‐M. Li , “Effects of Enhanced Recovery After Surgery Nursing Program on the Surgical Site Wound Infection and Postoperative Complications in Patients of Gastric Cancer: A Meta‐Analysis,” International Wound Journal 21, no. 2 (2024): e14687, 10.1111/iwj.14687.PMC1131054439118459

The above article, published online on 31 January 2024, in Wiley Online Library (http://onlinelibrary.wiley.com/), has been retracted by agreement between the journal Editor in Chief, Professor Keith Harding; and John Wiley & Sons Ltd. Following an investigation by the publisher, all parties have concluded that this article was accepted solely on the basis of a compromised peer review process. The editors have therefore decided to retract the article. The authors did not respond to our notice regarding the retraction.

